# Draft Genome Sequence of *Pantoea anthophila* Strain 11-2 from Hypersaline Lake Laysan, Hawaii

**DOI:** 10.1128/genomeA.00321-15

**Published:** 2015-05-14

**Authors:** Xuehua Wan, Shaobin Hou, Nolwenn Phan, Jennifer S. Malone Moss, Stuart P. Donachie, Maqsudul Alam

**Affiliations:** a Advanced Studies in Genomics, Proteomics and Bioinformatics, University of Hawaii, Honolulu, Hawaii, USA; b Department of Microbiology, University of Hawaii, Honolulu, Hawaii, USA

## Abstract

Most *Pantoea* spp. have been isolated from plant sources or clinical samples. However, we cultivated *Pantoea anthophila* 11-2 from hypersaline water from the lake on Laysan, Northwestern Hawaiian Islands. Draft genome sequencing of 11-2 provides a molecular basis for studies in evolution and pathogenicity in *Pantoea* spp.

## GENOME ANNOUNCEMENT

*Pantoea* spp. are Gram-negative bacteria generally isolated from agricultural or clinical samples, and marine sediment (1–3). Strain 11-2 from hypersaline Lake Laysan shared 100% nucleotide identity in its 16S rRNA gene with the 16S rRNA gene in *Pantoea anthophila* BD 871^T^ (LMG 2558) from *Impatiens balsamina* (4–6). We report the draft genome sequence of *P. anthophila* 11-2.

Shotgun and 8-kb-span paired-end libraries prepared and sequenced in the Roche 454 GS FLX+ platform generated 126.7 Mb of shotgun sequences and 116.3 Mb of 8-kb-span paired-end sequences, providing ~50× genome coverage. Assembly was performed in Newbler 2.8 in a two-step strategy. Shotgun reads were assembled into contigs, after which paired-end reads were added to build 5 scaffolds containing 4,609,867 bp (*N*_50_ = 3,885,396 bp) and 16 contigs containing 4,600,679 bp assembled in the 5 scaffolds. Gap regions in scaffolds were estimated to cover ~9 kb. The genome’s G+C content is 56.8%.

The genome was annotated in the NCBI Prokaryotic Genome Annotation Pipeline (PGAP) (7) and the Rapid Annotation Using Subsystem Technology (RAST) server (8, 9). PGAP identified 4,069 protein-coding open reading frames, 67 tRNA coding regions, and 15 rRNA coding regions. RAST identified 502 function-related subsystems and 7 phage components, including 3 phage baseplate proteins, 2 phage packaging machineries, and 1 each of phage tail protein and phage tail fiber protein. Six virulence regulation components were also identified in the BarA-UvrY two-component regulatory system. Ten proteins in the type III secretion system (T3SS) apparatus, and 64 T3SS-related transcription regulators were predicted using BLASTp against the T3SS database (T3DB) (E < 1e^−10^) (10). Eleven diguanylate cyclases and nine diguanylate phosphodiesterases (E < 1e^−10^) were predicted, which synthesize and degrade the ubiquitous second messenger, cyclic-di-GMP, involved in bacterial morphology and biofilm formation. The N-terminal PAS domain and GAF domain sensors of several diguanylate cyclases and diguanylate phosphodiesterases were predicted by the SMART server (11). The draft genome sequence reported here provides a molecular basis for studies in pathogenicity and evolution of *Pantoea* spp.

### Nucleotide sequence accession numbers.

This whole-genome shotgun project has been deposited at DDBJ/EMBL/GenBank under the accession number JXXL00000000. The version described in this paper is version JXXL01000000.
